# Helical inchworming: a novel translocation mechanism for a ring ATPase

**DOI:** 10.1007/s12551-021-00883-w

**Published:** 2021-11-24

**Authors:** Alexander B. Tong, Carlos Bustamante

**Affiliations:** 1 Jason L. Choy Laboratory of Single-Molecule Biophysics, University of California, Berkeley, CA USA; 2 Institute for Quantitative Biosciences-QB3, University of California, Berkeley, Berkeley, CA USA; 3 Chemistry Graduate Group, University of California, Berkeley, Berkeley, CA USA; 4 Department of Molecular and Cell Biology, University of California, Berkeley, Berkeley, CA USA; 5 Department of Chemistry, University of California, Berkeley, Berkeley, CA USA; 6 Department of Physics, University of California, Berkeley, Berkeley, CA USA; 7 Howard Hughes Medical Institute, University of California, Berkeley, Berkeley, CA USA; 8 Kavli Energy Nanoscience Institute, University of California, Berkeley, Berkeley, CA USA

**Keywords:** Ring atpase, Translocase, Optical tweezers, Cryo-electron microscopy

## Abstract

Ring ATPases perform a variety of tasks in the cell. Their function involves complex communication and coordination among the often identical subunits. Translocases in this group are of particular interest as they involve both chemical and mechanical actions in their operation. We study the DNA packaging motor of bacteriophage φ29, and using single-molecule optical tweezers and single-particle cryo-electron microscopy, have discovered a novel translocation mechanism for a molecular motor.

Multimeric ring ATPases encompass a wide family of proteins whose members carry out a plethora of different tasks in the cell (Snider et al. 2008). Their study poses interesting questions about how the individual subunits of these systems communicate and coordinate with each other throughout their mechanochemical cycle. Moreover, because of their common phylogeny, these questions and their answers are likely to be applicable across the group as a whole (Erzberger and Berger 2006). One such enzyme is the DNA packaging motor of bacteriophage φ29, a ring ATPase that displays impressive coordination among its subunits and which we have been studying using advanced single molecule biophysical methods such as optical tweezers. Moreover, the parallel use of advanced single-particle cryo-electron microscopy has permitted the collection of high-quality, substrate-engaged maps of the φ29 ATPase without the imposition of symmetry. We have combined the information gleaned from these two approaches to formulate a novel translocation mechanism for this molecular motor.

The DNA packaging motor of bacteriophage φ29 is a homopentameric ring ATPase that uses the energy of ATP hydrolysis to package its genome during viral assembly. Using optical tweezers, we have studied its translocation and have come to understand its mechanochemical cycle comprising a “dwell-burst” mechanism in which all subunits exchange ATP during a “dwell” or idle phase and a “burst” phase during which the ATPs of all five subunits are sequentially hydrolyzed to translocate 10 bp of DNA in four steps (Moffitt et al. 2009). We showed previously that the 10 bp burst is made of four steps of 2.5 bp each, with the remaining subunit and that one of the five subunits does not perform a mechanical task but rather a regulatory one (Chistol et al. 2012). Since 10 bp is very close to the periodicity of dsDNA (10.4 bp), we wondered whether the size of the burst is determined by the periodicity of the substrate being internalized in the capsid. How might the motor adapt to a polymer of differing helical periodicity? To this end, we tested the ability of the motor to package dsRNA and DNA:RNA hybrids which, surprisingly, were tolerated by the motor. Our results show that the amount of substrate translocated during the burst on these alternative substrates is reduced to match the substrates’ shorter helical periodicities (Castillo et al. 2021). Significantly, we find that the motor reduces its burst size with dsRNA and DNA:RNA hybrids by conserving the step size it uses for dsDNA during the first three steps and by shortening the fourth, rather than evenly reducing the size of all four.

In a parallel development, our collaborators solved the structure of the substrate-bound, ATP-full form of the motor using cryo-EM and found that the motor adopts a lock-washer structure that follows one strand of the DNA (the tracking strand) and spans one period (Woodson et al. 2021). What is then the mechanism of translocation of the bacteriophage φ29 packaging motor? Combining these structural results with the biophysical ones, we note that, if the lock-washer shape were to be maintained throughout the translocation phase or burst, the motor would lose grip of its substrate after just one of its four steps, as the helix of the motor would become unaligned with the helix of the substrate. This situation would not permit the motor to generate the 60 pN of force against which it can translocate in our single molecule assays (Chemla et al. 2005). Hence, we propose that the motor cycles between the lock-washer architecture and a planar one, which allows constant contact with the DNA, in a translocation mechanism that we term the helical inchworm model (Fig. 1a). Here we first describe the operation of the motor when packaging its normal substrate, dsDNA. At the beginning of the dwell phase, the motor is saturated with ADP and has adopted a planar configuration. In this state, only the regulatory or special subunit contacts a DNA phosphate, a contact that has both load-bearing and regulatory functions. As each subunit successively exchanges its ADP for ATP during the dwell, the ring opens in a step-wise manner until it is fully bound to ATP, attaining a lock-washer configuration. During this “opening” process, the subunits contact successive phosphates in the tracking strand of the dsDNA, conforming its lock-washer structure to the periodicity of the substrate. At this point, the motor has the strongest grip on the substrate as its subunits make electrostatic contacts with phosphates of the double helix. To start the burst, the special subunit hydrolyzes its ATP first, signaling the mechanical subunits to do the same in a sequential and ordinal fashion. In this process, phosphate release causes translocation via ring closing. At the end of this process, the motor is again in a planar state and the dwell can begin again. This model is consistent with the previous observations that identify the DNA phosphates as the moiety upon which the motor grips the substrate (Aathavan et al. 2009), and the higher grip of the motor when ATP bound as seen in other viral translocases (Ordyan et al. 2018).

**Fig. 1:**
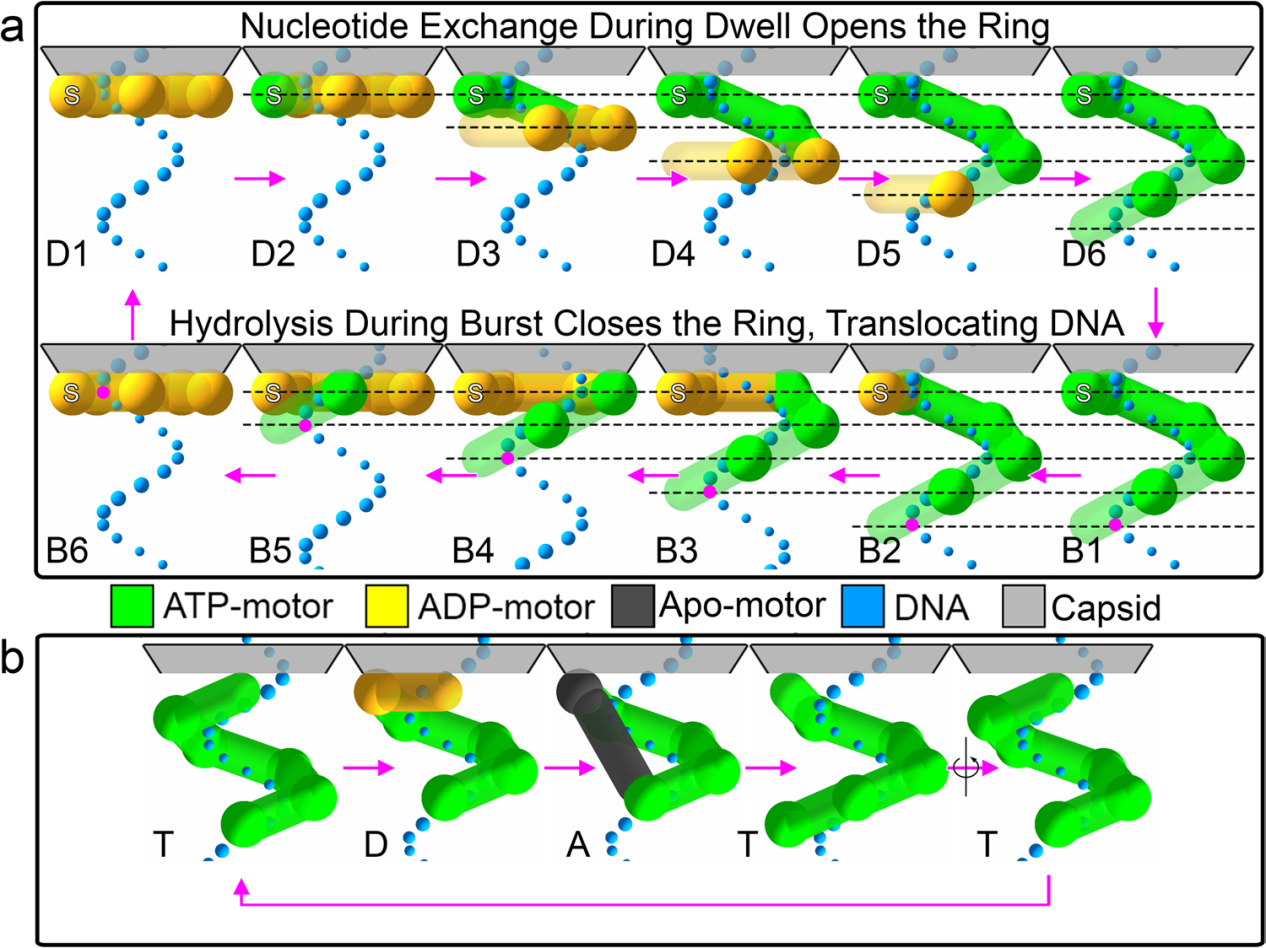
Mechanisms for lock-washer ring ATPase translocases. Shown above are cartoons of translocation mechanisms for a pentameric DNA packaging motor. The substrate is shown as a spiral of blue spheres, representing the phosphates of one strand of the DNA. The motor is represented by an assembly of larger spheres, colored by nucleotide state (green/yellow/black for ATP/ADP/apo, respectively) and the subunit connectivity is depicted by cylinders. The capsid is shown in gray above the motors, and the direction of packaging is towards the capsid. **a** In the helical inchworm mechanism, the motor first exchanges ADP for ATP while the ring opens to span one pitch of the DNA (D1–D6). Then, hydrolysis in the special subunit (marked with an S) causes the hydrolysis cascade (B1–B2), translocating 2.5 bp of DNA in four steps (B2–B6). A phosphate of the DNA is colored purple and dotted lines 2.5 bp apart are drawn as a guide. **b** In the hand-over-hand mechanism, we start from a fully ATP-bound motor (T). Hydrolysis of the uppermost subunit translocates 2 bp of DNA (D). Subsequent nucleotide exchange of this subunit causes it to relocate to the bottom of the ring (A, T). Now the cycle restarts, as the current state can be related to the original one via a rotation

On the other substrates, the motor has more difficulty to conform its lock-washer structure to their shorter helical periods. Moreover, this shorter distance means that, during ring opening, the last subunit grabs its phosphate in the substrate before it can completely open. This smaller cocking of the last subunit eventually results in a fourth translocation step during the burst that is smaller than those of the other three other subunits (which are similar to those made by the motor on dsDNA, as is experimentally observed). This model provides a mechanism by which the motor can “measure” and adapt its burst to the size of its substrate’s periodicity.

The presiding model for most of the other ring ATPase translocases that have been also shown to adopt lock-washer structures is the “hand over hand” mechanism. It differs from the helical inchworm model in that, instead of cycling between planar and helical architectures, the motor instead maintains its lock-washer architecture and subunits “jump” the gap in the ring as it translocates its substrate (Fig. 1b). These hand-over-hand motors have been proposed for ring ATPases that operate on disordered substrates, such as polypeptides or ssDNA on which they impose their helical geometry during translocation (Gao et al. 2019; de la Peña et al. 2018). The φ29 DNA packaging motor, however, must package a substrate possessing a pre-existing helical structure and it must be capable to adapt to the shortened helical periods of DNA:RNA hybrid and dsRNA. We liken the difference to that of a person climbing a rope versus a ladder. In the former case, one must deform the rope in order to better grip it with one’s hands and legs, while in the latter the grip points are predetermined by the rungs of the ladder. In the hand-over-hand mechanism, the helical structure of the motor imposes helicity onto the substrate in order to maintain grip on it, while in the helical inchworm mechanism, the motor instead alters its operation to fit the pre-existing structure of the substrate, as a climber would do with a ladder possessing closer or more separated rungs. The rope/ladder metaphor also explains the higher forces (60 pN) that the φ29 translocase can exert compared to hand-over-hand protein translocases (15 pN) (Maillard et al. 2011) in terms of the relative grip strength of a climber that uses a ladder instead of a rope.
